# Assessment of rational use of antimicrobials: a cross-sectional study among people of Nepal

**DOI:** 10.1097/MS9.0000000000000925

**Published:** 2023-05-24

**Authors:** Sitaram Khadka, Sulochana Khadka, Gopal K. Yadav, Arun Sharma, Santoshi Giri, Rinku Joshi, Kapil Amgain

**Affiliations:** a Shree Birendra Hospital, Nepalese Army Institute of Health Sciences; b Nepalese Army Institute of Health Sciences, College of Medicine; c Narayani Hospital, Ministry of Health and Population, Birgunj; d Nepal Public Health Research and Development Center, Kathmandu; e Karnali Academy of Health Sciences, Jumla, Nepal

**Keywords:** antimicrobials, developing countries, educational status, Nepal, policy

## Abstract

Inappropriate antimicrobial use leading to antimicrobial resistance is a matter of public health concern globally. This research was conducted with the objective of preventing antimicrobial misuse across knowledge, behavior, and practice domains among the general people of Nepal.

**Methods:**

It was a cross-sectional survey conducted among 385 participants visiting tertiary care center from all over Nepal from February 2022 to May 2022. Modified Bloom’s cut-off point was utilized to categorize the participants’ overall knowledge, behavior, and practice. The chi-square (*χ* ^2^) test and odds ratio (OR) using binary logistic regression at 95% CI and Spearman’s rank correlation coefficient test (*r*) were calculated wherever appropriate.

**Results:**

More than three-fifths of the participants (248, 64.42%) demonstrated good behavior, whereas less than half of the participants showed good knowledge (137, 35.58%) and practice (161, 41.82%) about rational use of antimicrobials. Health professionals had higher knowledge (OR: 1.07, 95% CI: 0.70–1.62) and good behavior (OR: 0.42, 95% CI: 0.27–0.64) than other professionals (*P*<0.05). Those with higher income [≥50 000 NRS (Nepalese rupees)] had good behavior (OR: 3.37, 95% CI: 1.65–6.87) and good practice (OR: 2.58, 95% CI: 1.47–4.50) scores than those with less monthly income (*P*<0.05). Similarly, higher educational degrees, viz. master’s and/or above, had good behavior (OR: 4.13, 95% CI: 2.62–6.49) and good practice scores (OR: 2.55, 95% CI: 1.68–3.87). Additionally, there were significant positive correlations between knowledge (K), behavior (B), and practice (P) scores (*r*=0.331 for K and B, *r*=0.259 for K and P, and *r*=0.618 for B and P, respectively; *P*<0.05).

**Conclusions:**

The findings imply the demand for effective legislature, strict enforcement of the drug act, and proper implementation of plans and policies to curb antimicrobials misuse. Lack of execution of existing laws and the unawareness of the public led to the extravagant use of antimicrobials.

## Introduction

Highlights

- Rational use of antimicrobials (RUM) is the need of the hour.
- Antimicrobial resistance (AMR) could be addressed by adopting the One Health approach.
- This study provides baseline data to know peoples’ perceptions about AMR in low-income and middle-income countries.
- Lack of execution of existing laws and people’s unawareness are liable for AMR.
- Training and seminars at the policy-making level are highly recommended for RUM.

The irrational use of antimicrobials is a global issue. The use of antimicrobials and their acquisition from pharmacies without prescription is on the rise in low-income and middle-income countries (LMICs)^1,2^. Inappropriate antimicrobial prescription is also widely reported all over the world, including the developed countries^3^. These practices consequently promote the irrational use of antimicrobials which has long-term effects on patients’ health^4^. The World Health Organization’s (WHO) Global Strategy for Containment of Antimicrobial Resistance outlines appropriate antimicrobial use as the cost-effective use of antimicrobials that maximizes clinical therapeutic effect whilst minimizing drug-related toxicity and development of antimicrobial resistance (AMR)^5^.

Antimicrobials, which are crucial for the treatment of varieties of infections, came into practice after Alexander Fleming discovered penicillin in 1928^6^. AMR can be attributed to irrational use of antimicrobials; unnecessary, suboptimal (duration, frequency, indication, dose, and dosage form), and extensive use of broad-spectrum antimicrobials^7,8^. AMR is one of the greatest threats to public health that is responsible for increased morbidity and mortality as well as augmented healthcare costs^9,10^. The WHO launched a Global Action Plan (GAP) in 2015 to initiate evidence-based prescribing through effective, rapid, and low-cost diagnostic tools to optimize the use of antimicrobials^4^. Nepal has also developed National Antimicrobials Resistance Containment Action Plan (NAP), 2016, on the basis of GAP^11^. However, its implementation is still a challenge as the majority of healthcare workers are unaware of this action plan, and its utilization is very low.

Despite these efforts, the volume of antimicrobial use is ever-increasing worldwide, especially in LMICs, with India in the first and China in the second position^12,13^. The incidence of AMR is more prevalent in LMICs due to poor enforcement of laws and a lack of substantial surveillance systems^14^. Nepal is no different. However, Nepal has made a significant effort for the rational use of medicines (RUM), especially antimicrobials. National Antimicrobials Treatment Guidelines, 2014 and Antimicrobial Resistance Containment Guideline, 2019, have been endorsed^15^. However, there is no sufficient surveillance system for tracking current antimicrobials use and its resistance pattern in Nepal.

Although individuals and institutions are making sporadic attempts in all domains of AMR, there is a lack of coordinated action. Current researches and published literatures are not sufficient enough to elucidate the current scenario. This research was carried out with the objective of assessing the knowledge, behavior, and practice of antimicrobials misuse among the study participants in the context of Nepal to understand the factors leading to the misuse of antimicrobials so that effective interventions can be implemented to promote rational drug therapy.

## Methods

### Ethics approval

The research was performed in accordance with the Declaration of Helsinki. The ethics approval was granted by the Institutional Review Committee (IRC) of the Nepalese Army Institute of Health Sciences, Kathmandu, Nepal (Reg. No 572). Informed e-consent to participate in the study was obtained from participants.

### Study design

A cross-sectional survey was carried out principally targeting the assessment of the antimicrobial use patterns in a random sample of the general population who visited a tertiary care center from all over Nepal. The convenience and snowball sampling methods were used for collecting data. The study was conducted in accordance with the STROCSS (strengthening the reporting of cohort, cross-sectional and case–control studies in surgery) 2021 criteria^16,^ Supplemental Digital Content 1, http://links.lww.com/MS9/A146. This research was registered in Research Registry (Researchregistry8637).

### Study setting

This study was carried out in a tertiary care center in Kathmandu, Nepal, from 14th February 2022 to 15th May 2022. The setting is Shree Birendra Hospital, Kathmandu, a multispecialty referral center where almost 1200 people every day from nooks and corners of the country visit for their treatment. Nepal is a landlocked country in South-East Asia situated between China and India. It has an area of 147 516 km^2^ and an estimated population of 29 192 480 as per the 2021 census^17^.

### Study population

**Figure 1:**
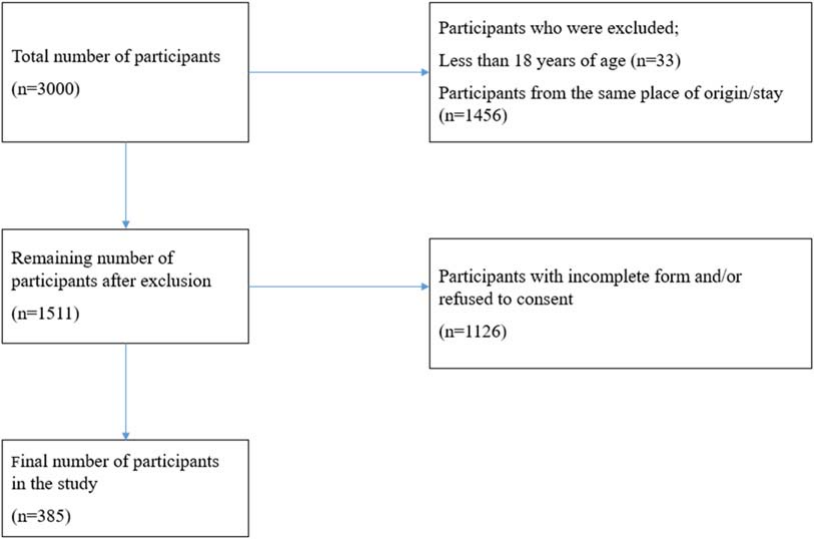
Flow diagram showing the selection of the participants.

**Figure 2:**
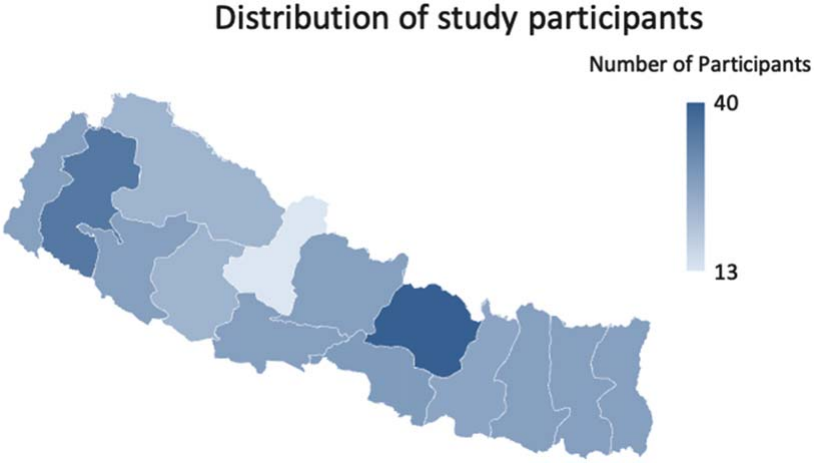
Map showing the distribution of participants.

A total of 385 participants were selected using convenience and snowball sampling methods. The patients, as well as their caregivers, visitors, and other people with whom they had contact, were also included as participants to cover people from all over the nation. The flow diagram depicting the participants’ inclusions and map showing the distribution of participants have been drawn below (Figs 1, 2).

### Study tool and data collection

A semi-structured questionnaire was developed in English as well as in the Nepali language comprising five sections; information and informed consent, demographics of participants, knowledge, behavior, and practices on antimicrobials use on a five-point Likert scale (strongly disagree, disagree, neutral, agree, strongly agree). The questionnaire was validated by the expert review team of the Nepalese Army Institute of Health Sciences, Kathmandu, Nepal. It was pretested on 20 participants, which were not included in the final data set. Then, the pretested and verified questionnaire was sent to the participants via different online media (Messenger, WhatsApp, Viber, etc.) in the form of Google Forms. The face-to-face data collection was also done in cases where the participants were not familiar or hesitant with online media.

### Data management and statistical analysis

The response to the online survey was extracted from google docs as Excel 2019 v16.0 (Microsoft, Washington, USA) and exported to IBM SPSS v21 (IBM, Armonk, New York) and MedCalc for Windows v 12.3.0 (MedCalc-Software, Mariakerke, Belgium) for the further analysis.

The knowledge and behaviors items consist of five questions (maximum score of 25), while the practice item consists of 10 questions (maximum score of 50). The statements which opposed the notion of knowledge, behaviors, and/or practices were graded 5 points for strongly disagree and 1 point for strongly agree, and accordingly rest responses of disagree, neutral/unsure, and agree were graded 4, 3, and 2 points in decremental order respectively. Similarly, the statements which supported the notion of knowledge, behavior, and/or practices were graded 1 point for strongly disagree and 5 points for strongly agree, and accordingly rest responses of disagree, neutral/unsure, and agree were graded 2, 3, and 4 points, respectively.

The total scores of knowledge, behavior, and practice were calculated and recoded into different categorical variables. The good [ ≥80%of25=20 for knowledge and behavior items and ≥80%of50=40 for practice items] and the moderate to poor group (<80%) were categorized for each knowledge, behaviors, and practice items based on modified Bloom’s cut-off criteria^16^. Sociodemographic characteristics of participants were presented as frequency and proportions. The chi-square (*χ* ^2^) test was used to test for group differences. For binary logistic regression analyses, the odds ratio (OR) was calculated at 95% confidence intervals (95% CI). Box plots were drawn for the distribution of knowledge, behavior, and practice scores based on education level and areas of work. Spearman’s rank correlation coefficient test was used to assess the relationships among the knowledge, behavior, and/or practice scores.

## Results

### Sociodemographic data

Maximum participants were of age below 40 years (360, 93.51%), and more than two-fifths were male (159, 41.30%). Most participants had educational qualifications of higher secondary and above level (365, 94.80%). More than one-fifth of the participants were from rural areas (80, 20.58%). More than three-fifths of respondents were health professionals (240, 62.34%). The majority of the participants, more than 80% (323, 83.89), had income less than 50 000 NRS (Nepalese rupees). Few participants were smokers (15, 3.89%). More than one-fifth had comorbid state (63, 16.36%) (Table 1).

**Table 1:** Sociodemographic characteristics of the participants (*N*=385).

Serial number	Characteristics	Frequency	Proportion (%)
1	Age		
	Below 40	360	93.51
	40–59	23	5.97
	60 and above	2	0.52
2	Sex		
	Male	159	41.30
	Female	226	58.70
3	Education		
	No formal education	4	1.04
	Up to secondary level	16	4.16
	Higher secondary level	175	45.45
	Bachelor level and above	190	49.35
4	Residency/area		
	Urban	305	79.22
	Rural	80	20.78
5	Occupation		
	Student	202	52.47
	Employed	143	37.14
	Self-employed/Private	19	4.94
	Not employed	21	5.45
6	Nature of work		
	Health professionals	240	62.34
	Others	145	37.67
7	Income		
	Less than 20 000	113	29.35
	20 000–49 999	210	54.54
	50 000–100 000	41	10.65
	More than 100 000	21	5.54
8	Smoking status		
	Yes	15	3.89
	No	370	96.10
9	Comorbidities		
	No	322	83.63
	Cardiovascular diseases	17	4.42
	Endocrine disorders	19	4.94
	Respiratory diseases	1	0.26
	Other diseases	26	6.75

### Knowledge assessment

The responses are reported in Supplementary Table 1 (Supplemental Digital Content 1, http://links.lww.com/MS9/A147). The median knowledge score was 18 (25th–75th percentiles: 17–20). More than one-third of the participants (137, 35.58%) had good knowledge regarding the RUM. The health professionals had 1.79 times higher odds of good knowledge than other professionals (OR 0.56, 95% CI 0.36–0.88) (Tables 2 and 3).

**Table 2:** Knowledge, behavior, and practice scores of the participants.

Serial number	Characteristics	Scores/values
1	Knowledge	
	Median (min–max)	18 (13–25)
	Q1–Q3	17–20
	Mean±SD	18.51±2.19
	Good knowledge	35.58% (137/385)
2	Behavior	
	Median (min–max)	21 (8–25)
	Q1–Q3	18–23
	Mean±SD	20.73±3.11
	Good behavior	64.42% (248/385)
3	Practice	
	Median (min–max)	38 (12–50)
	Q1–Q3	34–43
	Mean±SD	37.73±6.93
	Good practice	41.82% (161/385)

**Table 3:** Factors affecting the knowledge of the participants about the rational use of antimicrobials.

		Knowledge		Binary logistic regression		
Serial number	Characteristics	Moderate to poor (%)	Good (%)	OR	95% CI	*P*
1	Age					
	<40 years	231 (64.17)	129 (35.83)	1 (Ref.)		
	≥40 years	17 (68.00)	8 (32.00)	0.84	0.35–2.01	0.699
2	Sex					
	Male	95 (59.75)	64 (40.25)	1 (Ref.)		
	Female	153 (67.70)	73 (32.30)	0.71	0.46–1.08	0.109
3	Education					
	Up to Bachelor	124 (63.59)	71 (36.41)	1 (Ref.)		
	Master and above	124 (65.26)	66 (34.74)	0.93	0.61–1.41	0.732
4	Residency/area					
	Urban	200 (65.57)	105 (34.43)	1 (Ref.)		
	Rural	48 (60.00)	32 (40.00)	1.27	0.77–2.11	0.354
5	Occupation					
	Not employed/Student	145 (65.02)	78 (34.98)	1 (Ref.)		
	Employed	103 (63.58)	59 (36.42)	1.07	0.70–1.62	0.770
6	Nature of work					
	Health professionals	143 (59.58)	97 (40.42)	1 (Ref.)		
	Others	105 (72.41)	40 (27.59)	0.56	0.36–0.88	0.011
7	Income					
	<50 000	213 (65.94)	110 (34.05)	1 (Ref.)		
	≥50 000	35 (56.45)	27 (43.55)	1.49	0.86–2.60	0.153
8	Smoking status					
	Yes	10 (66.67)	5 (33.33)	1 (Ref.)		
	No	238 (64.32)	132 (35.68)	1.11	0.37–3.31	0.853
9	Comorbidities					
	Yes	43 (68.25)	20 (31.75)	1 (Ref.)		
	No	205 (63.66)	117 (36.34)	1.23	0.69–2.19	0.487

### Behavior assessment

The responses are reported in Supplementary Table 2 (Supplemental Digital Content 1, http://links.lww.com/MS9/A147). The median behavior score was 21 (25th–75th percentiles: 18–23). More than three-fifths of the participants (248, 64.42%) had good behavior regarding the RUM. The participants with an education master’s degree and/or above had more than two times higher odds of good behavior (OR 2.55, 95% CI 1.68–3.87). Employed participants had more than two times higher odds of good behavior (OR 2.90, 95% CI 1.84–4.57) than non-employed or students. The health professionals had 2.38 times higher odds of good behavior than other professionals (OR 0.42, 95% CI 0.27–0.64). Similarly, participants with an income of more than 50 000 NRS had 3.37 times higher odds of good behavior (OR 3.37, 95% CI 1.65–6.87) (Tables 2, 4).

**Table 4:** Factors affecting the behavior of the participants about the rational use of antimicrobials.

		Behavior		Binary logistic regression		
SN	Characteristics	Moderate to poor (%)	Good (%)	OR	95% CI	*P*
1	Age					
	<40 years	124 (34.44)	236 (65.56)	1 (Ref.)		
	≥40 years	13 (52.00)	12 (48.00)	0.49	0.22–1.10	0.076
2	Sex					
	Male	52 (32.70)	107 (6.30)	1 (Ref.)		
	Female	85 (37.61)	141 (62.39)	0.81	0.53–1.24	0.322
3	Education					
	Up to Bachelor	99 (50.77)	96 (49.23)	1 (Ref.)		
	Master and above	38 (20.00)	152 (80.00)	4.13	2.62–6.49	<0.001
4	Residency/area					
	Urban	108 (35.41)	197 (64.59)	1 (Ref.)		
	Rural	29 (36.25)	51 (63.75)	0.96	0.58–1.61	0.889
5	Occupation					
	Not employed/Student	101 (45.29)	122 (54.71)	1 (Ref.)		
	Employed	36 (22.22)	126 (77.78)	2.90	1.84–4.57	<0.001
6	Nature of work					
	Health professionals	67 (27.92)	173 (72.08)	1 (Ref.)		
	Others	70 (48.28)	75 (51.72)	0.42	0.27–0.64	<0.001
7	Income					
	<50 000	127 (39.32)	196 (60.68)	1 (Ref.)		
	≥50 000	10 (16.13)	52 (83.87)	3.37	1.65–6.87	<0.001
8	Smoking status					
	Yes	7 (46.67)	8 (53.33)	1 (Ref.)		
	No	130 (35.14)	240 (64.86)	1.62	0.57–4.56	0.360
9	Comorbidities					
	Yes	16 (25.40)	47 (74.60)	1 (Ref.)		
	No	121 (37.58)	201 (62.42)	0.57	0.31–1.04	0.065

### Practice assessment

The responses are reported in Supplementary Table 3 (Supplemental Digital Content 1, http://links.lww.com/MS9/A147). The median practice score was 38 (25th–75th percentiles: 34–43). More than two-fifth of the participants (161, 41.82%) demonstrated good practice regarding the RUM. The participants with an education master’s degree and/or above had more than four times higher odds of good practice (OR 2.55, 95% CI 1.68–3.87). Employed participants had more than two times higher odds of good practice (OR 2.56, 95% CI 1.68–3.88) than non-employed or students. Similarly, participants with an income of more than 50 000 NRS had 2.58 times higher odds of good practice (OR 2.58, 95% CI 1.47–4.50) (Tables 2 and 5).

**Table 5:** Factors affecting the practice of the participants regarding the rational use of antimicrobials.

		Practice		Binary logistic regression		
Serial numbers	Characteristics	Moderate to poor (%)	Good (%)	OR	95% CI	*P*
1	Age					
	<40 years	211 (58.61)	149 (41.39)	1 (Ref.)		
	≥40 years	13 (52.00)	12 (48.00)	1.31	0.58–2.95	0.517
2	Sex					
	Male	89 (55.97)	70 (44.03)	1 (Ref.)		
	Female	135 (59.73)	91 (40.27)	0.86	0.57–1.29	0.462
3	Education					
	Up to Bachelor	135 (69.23)	60 (30.77)	1 (Ref.)		
	Master and above	89 (46.84)	101 (53.16)	2.55	1.68–3.87	<0.001
4	Residency/area					
	Urban	173 (56.72)	132 (43.28)	1 (Ref.)		
	Rural	51 (63.75)	29 (36.25)	0.75	0.45–1.24	0.257
5	Occupation					
	Not employed/Student	151 (67.71)	72 (32.29)	1 (Ref.)		
	Employed	73 (45.06)	89 (54.94)	2.56	1.68–3.88	<0.001
6	Nature of work					
	Health professionals	132 (55.00)	108 (45.00)	1 (Ref.)		
	Others	92 (63.45)	53 (36.55)	0.70	0.46–1.08	0.103
7	Income					
	<50 000	200 (61.92)	123 (38.08)	1 (Ref.)		
	≥50 000	24 (38.71)	38 (61.29)	2.58	1.47–4.50	<0.001
8	Smoking status					
	Yes	7 (46.67)	8 (53.33)	1 (Ref.)		
	No	217 (58.65)	153 (41.35)	0.62	0.22–1.74	0.356
9	Comorbidities					
	Yes	33 (52.38)	30 (47.62)	1 (Ref.)		
	No	191 (59.32)	131 (40.68)	0.75	0.44–1.30	0.307

### Distribution of knowledge, behavior, and practice scores based on the education level

**Figure 3:**
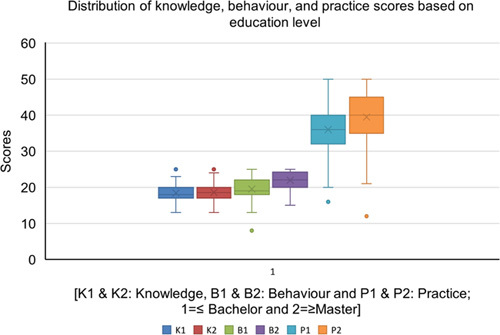
Box plots of the distribution of knowledge, behavior, and practice scores based on education level.

Those participants with master’s degrees and above educational levels had higher median behavior and practice scores as compared to those with lower educational levels. However, there was no statistical difference across both groups in terms of knowledge, behavior, and practice scores (Fig. 3).

### Distribution of knowledge, behavior, and practice scores based on areas of work

**Figure 4:**
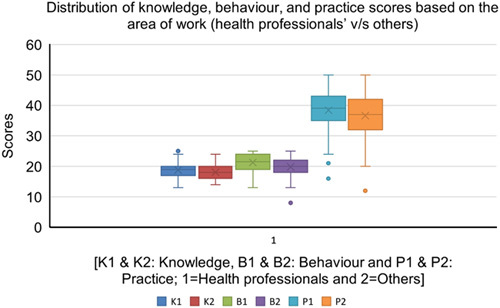
Box plots of the distribution of knowledge, behavior, and practice scores based on the area of work (health professionals versus others).

There was no statistical difference across both groups (health professionals versus others) in terms of knowledge, behavior, and practice scores (Fig. 4).

### Correlation between scores

There was a significant correlation between knowledge and behavior scores [Spearman’s rho (*ρ*): 0.331; *P*≤0.001, 95% CI=0.24–0.42]. Similarly, the knowledge and practice scores (Spearman’s *ρ*: 0.259; *P*<0.001, 95% CI=0.16–0.35) and behavior and practice scores were positively correlated (Spearman’s *ρ*: 0.618; *P*≤0.001, 95% CI=0.55–0.68).

## Discussion

Accumulative risk due to AMR caused by irrational use of antimicrobials is a global health concern. Such risks lead to health and economic ramifications, including preventable deaths, increased healthcare expenditure, and higher levels of healthcare overheads^18^. ‘Targeted spectrum’ antimicrobials used in the appropriate dose and duration can prevent the evolving AMR^19^. In our study, different factors such as age, gender, occupation, nature of work, income, residency, and education were evaluated to assess the knowledge, behavior, and practice of antimicrobial misuse. More than three-fifths of the participants demonstrated good behavior, whereas less than half of the participants showed good knowledge and practice. A study performed in Saudi Arabia showed insufficient knowledge regarding safe antimicrobial use^20^. However, some studies done in China and UAE demonstrated better knowledge, attitude, and practice of antimicrobial use in a specific group of people like medical students, which is in order with our findings where health professionals were better in terms of knowledge, attitude, and practice than other professionals (CI 0.36–0.88, *P*<0.011)^21,22^.

The present study showed that about one-third of participants believed that antimicrobials could be obtained from a pharmacy without a doctor’s prescription, like other over-the-counter (OTC) drugs. Less than one-fifth of study participants took antimicrobials without a prescription. A similar finding was seen in undergraduate students of Nigeria, among the metropolitan people of Thailand, people of South India, and Northwest Ethiopia^23–26^. A meta-analysis on the prevalence of antimicrobials self-medication in developing countries was found to be in a higher range than our finding^27^. A study done in LMICs like Eritrea showed the extent of dispensing antimicrobials without prescription up to 87%^28^. This shows that obtaining antimicrobials without a prescription is a common practice all over the world. The reason could be a lack of awareness on the importance of using prescribed medication and a lack of surveillance of local pharmacies on the prescription of antimicrobials.

In our study, about one-third of the respondents stopped taking antimicrobials as soon as the symptoms subsided. The result is similar to the study in Northwest Ethiopia and higher in Saudi Arabia^20,26^. Usually, patients approach directly to pharmacy/pharmacists for resolving their medical issues, and they exert great pressure on such professionals to obtain antimicrobials without a prescription^29^. Thus, pharmacists in the community can play a role as first-line health professionals in effective patient counseling and public awareness regarding the use of prescription antimicrobials and the adverse effects of antimicrobial misuse.

In our study, less than one-fifth of respondents requested physicians to prescribe medications without a culture report. A similar study in Pakistan had a higher percentage (38.1%) of study participants who thought that there was no need for culture analysis before dispensing antimicrobials^30^. Although policy guidelines demand the use of antimicrobials based on the identification of causative agents, the empirical and OTC use of antimicrobials is high in practice^31^. Performing culture is crucial to understand the resistance pattern in the patient. Knowing the trends in sensitivity and resistance patterns through culture and sensitivity tests can assist in making better judgments about how to address potential resistance^32,33^.

Knowledge regarding the use of antimicrobials against minor ailments like the common cold, flu, and cough was seemingly found to be poor as one-third of the respondents thought of using antimicrobials for such ailments while less than one-third actually took them, which is in order with the results of a study done in Kuwait, Jordan, and the U.K.^34–36^. This is probably due to inadequate knowledge of antimicrobial use. Accessibility of antimicrobials is also a major concern in many countries worldwide, especially LMICs^37^. The findings of our study concurred with the statement as almost half of the respondents mentioned that they had taken antimicrobials of a similar group because the prescribed one was not available. The reasons for inaccessibility are attributed to the very low economic status of a large number of nations, inappropriate use, high cost of the most recent and efficacious antimicrobials, extensive OTC usage, an increasing number of counterfeit drugs, and a dramatic increase in AMR^38^.

Of the respondents, about one-third took low doses of antimicrobials due to fear of side effects in this study, and about one-fourth took the leftover antimicrobials, contrary to which a study done in Germany presented that 88.7% received advice before taking prescribed antimicrobials^39^. However, expired drugs are widespread, mainly in LMICs, where many types of drugs are sold without prescriptions^25^. It is healthcare professionals’ responsibility to provide proper counseling on usages such as dose, treatment course, and the harmful effects of misuse^40^. However, it is lacking in LMICs like Nepal, owing to the doctor’s less time contribution to the patient due to the low doctor-to-patient ratio. Rampant antimicrobials use and ignorance of people about the knowledge of the course of antimicrobials, their side effects, and standard acceptable dosage limits are the potential reasons for inappropriate or incorrect treatment, which can lead to AMR issues and increased morbidity^41^.

Our study findings showed a significant association between the nature of work and knowledge and behavior regarding antimicrobial use among respondents, which corresponded with the study done in Nepal and Hong Kong^42,43^. The knowledge and practice of antimicrobial use and AMR were significantly dependent on the educational level of the respondents, which aligns with the findings of a study performed in Lithuania where people with an educational level of a college degree and above had better knowledge, more appropriate behaviors, and better practices^44^. Similarly, the level of income was significantly and synonymously associated with the behavior and practice of antimicrobial use in our study, which contrasts with a study performed recently but similar to the study in Saudi Arabia^45,46^. The remaining demographic variables did not significantly influence the knowledge, behavior, and practice of antimicrobial use. The exact reason for this is currently not clear, but it can be postulated that the different geographic locations, healthcare regulations, and policies of the nation may be a contributing factor, although future research is needed to investigate.

An important concern for AMR is the irrational use of antimicrobials for plants and food animals. Antimicrobials use in various industries; for instance, in the United States, agriculture, farming, and aquaculture account for around 80% of antimicrobials usage^47^. In LMICs, antimicrobials are frequently employed against rice to combat mites and insects that are not affected by those antimicrobials^48^. The potential hazard to human health arising from incorrect antimicrobial use in food animals is also high^49^. Hence, the agricultural use of antimicrobials should also be acknowledged as one of the key factors in the emergence of resistant microorganisms.

AMR could be addressed by adopting the One Health (OH) approach that brings together humans, animals, and the environmental sector. The government of Nepal has also adopted the approach as highlighted by GAP^11,50^. However, challenges include a lack of separate institutional setup, lack of awareness among professionals in human and animal health, and environment sectors, unclear job and responsibilities, and regulatory mechanisms^51^. Training and seminars from the policy-making level are highly recommended. Public dissemination of information using television, newspapers, and social media is an effective method of increasing health literacy.

### Strengths and limitations

Our findings can be considered in the context of several limitations. First, the results contain self-reported, online reports which may not reflect the actual behavior. Second, the response to the survey was based on the author’s network, and as such, they might ignore valuable comments from other people who had not been surveyed. Third, most of the study participants had a high academic level. Fourth, most participants belonged to urban areas with better access to information on antimicrobials. However, our findings can provide helpful information for determining antimicrobial use awareness.

## Conclusion

The findings of this study may serve as baseline data to understand people’s perceptions regarding AMR in LMICs. This study recommends effective legislature – strict enforcement of the drug act and proper implementation of other relevant plans and policies. Studies focusing on effective public education and awareness are highly recommended. Further studies are also necessary to evaluate the effects of awareness programs on antimicrobial use that help contain AMR.

## Ethics approval

The ethical approval was obtained from the Institutional Review Committee of the Nepalese Army Institute of Health Sciences (IRC-NAIHS) (Reg No: 572). All procedures performed in studies involving human participants were in accordance with the ethical standards of IRC of NAIHS and with the Declaration of Helsinki, 1964, and its later amendments or comparable ethical standards.

## Consent

All the participants were made aware of their voluntary participation and confidentiality prior to the data collection. Informed consent to participate in the study was obtained from participants.

## Sources of funding

This article did not receive any kind of grant.

## Author contribution

Sitaram K.: conceptualization, investigation, methodology, and writing – original draft; Sulochana K. and G.K.Y.: data curation, formal analysis, investigation, writing – original draft; A.S.: conceptualization, project administration, and writing – review and editing; Santoshi G., R.J., and K.A.: visualization, writing – original draft, review, and editing.

## Conflicts of interest disclosure

There are no conflicts of interest.

## Research registration unique identifying number (UIN)

1. Name of the registry: Research Registry.
2. Unique identifying number or registration ID: Researchregistry8637.
3. Hyperlink to specific registration (must be publicly accessible and will be checked): https://www.researchregistry.com/register-now#home/registrationdetails/63cab3e69aa88a0012789649/

## Guarantor

Sitaram Khadka, Address: Shree Birendra Hospital, Nepalese Army Institute of Health Sciences, Kathmandu, Nepal, Tel.: +977 98510 77589, E-mail: sitaram.khadka@naihs.edu.np

## Data availability statement

The data are available through the corresponding author upon reasonable request.

## Peer review file

All authors agree to the publication of the peer review file for transparency.

## Provenance and peer review

Not commissioned, externally peer-reviewed.

## Supplementary Material
