# Supplementary material for: Assessment of rational use of antimicrobials: a cross-sectional study among people of Nepal
Source: Ann Med Surg (Lond). 2023 May 24;85(7):3372–80. doi: 10.1097/MS9.0000000000000925 (PMC10328652; doi:10.1097/MS9.0000000000000925)
Supplement: Supplementary file 1 [file ms9-85-3372-s001.docx]

| **Research and reporting methodology** |  | |  |
| --- | --- | --- | --- |
| Revised **Standards for QUality Improvement Reporting Excellence** (**SQUIRE 2.0**) publication guidelines |  | |  |
|  |  | |  |
| **Notes to authors** |  | |  |
| ▸ The SQUIRE guidelines provide a framework for reporting new knowledge about how to improve healthcare. |  | |  |
| ▸ The SQUIRE guidelines are intended for reports that describe system level work to improve the quality, safety and value of healthcare, and used methods to establish that observed outcomes were due to the intervention(s). |  | |  |
| ▸ A range of approaches exists for improving healthcare. SQUIRE may be adapted for reporting any of these. |  | |  |
| ▸ Authors should consider every SQUIRE item, but it may be inappropriate or unnecessary to include every SQUIRE element in a particular manuscript. |  | |  |
| ▸ The SQUIRE glossary contains definitions of many of the key words in SQUIRE. |  | |  |
| ▸ The explanation and elaboration document provide specific examples of well-written SQUIRE items and an in-depth explanation of each item. |  | |  |
| ▸ Please cite SQUIRE when it is used to write a manuscript. |  | |  |
|  |  | |  |
| **Text section and item name** | | **Page/line no(s).** | |
|  | | **info is located** | |
| **Title and abstract** | |  | |
| 1. **Title** | | **Page 1** (Line 1-2) | |
| Indicate that the manuscript concerns an initiative to improve healthcare (broadly defined to include the quality, safety, effectiveness, patient-centeredness, timeliness, cost, efficiency and equity of healthcare). | | Page 1 (Line 1-2) | |
|  | |  | |
| 2. **Abstract** | | **Page 1** | |
| a. Provide adequate information to aid in searching and indexing. | | Page 1 (Line 26 and 27) | |
| b. Summaries all key information from various sections of the text using the abstract format of the intended publication or a structured summary such as: background, local problem, methods, interventions, results, conclusions. | | Page 1 (Line 5 to Line 25) | |
|  | |  | |
| **Introduction: Why did you start?** | | **Page 1 (Line 29) to Page2 (Line 58)** | |
| 3. **Problem description** - Nature and significance of the local problem. | | **Page 1** (Line 29) to **Page 2** (Line 41) | |
| 4. **Available knowledge** - Summary of what is currently known about the problem, including relevant previous studies. | | **Page 2** (Line 51 to 82) | |
| 5. **Rationale** - Informal or formal frameworks, models, concepts and/or theories used to explain the problem, any reasons or assumptions that were used to develop the intervention(s) and reasons why the intervention(s) was expected to work | | **Page 2** (Line 83) to Page 3 (Line 102) | |
| 6. **Specific aims** - Purpose of the project and of this report. | | **Page 3** (Line 96 to 102) | |
|  | |  | |
| **Methods: What did you do?** | | **Page 3** (Line 104) to **Page 7** (Line 252) | |
| 7. **Context** - Contextual elements considered important at the outset of introducing the intervention(s). | | **Page 3** (Line 94-99) | |

| 8. **Intervention(s)** | Not applicable |
| --- | --- |
| a. Description of the intervention(s) in sufficient detail that others could reproduce it. |  |
| b. Specifics of the team involved in the work. |  |
| 9. **Study of the intervention(s)** | Not applicable |
| a. Approach chosen for assessing the impact of the intervention(s). |  |
| b. Approach used to establish whether the observed outcomes were due to the intervention(s). |  |
| 10. **Measures** | √ |
| a. Measures chosen for studying processes and outcomes of the intervention(s), including rationale for choosing them, their operational definitions and their validity and reliability. | **Page 3** (Line 105) to **Page 7** (Line 252) |
| b. Description of the approach to the ongoing assessment of contextual elements that contributed to the success, failure, efficiency and cost. | Page 3 (Line 105) to Page 7 (Line 252) |
| c. Methods employed for assessing completeness and accuracy of data. | Page 5 (Line 170) to Page 6 (Line 207) |
| 11. **Analysis** | √ |
| a. Qualitative and quantitative methods used to draw inferences from the data. | **Page 3** (Line 105) to **Page 7** (Line 251) |
| b. Methods for understanding variation within the data, including the effects of time as a variable. | **Page 5** (Line 171 to 193) |
| 12. **Ethical considerations** - Ethical aspects of implementing and studying the intervention(s) and how they were addressed, including, but not limited to, formal ethics review and potential conflict(s) of interest. | Not applicable |
|  |  |
| **Results: What did you find?** | **Page 7** (Line 254) to **Page 15** (Line 555) |
| 13. **Results** | √ |
| a. Initial steps of the intervention(s) and their evolution over time (eg, time-line diagram, flow chart or table), including modifications made to the intervention during the project. | **Page 7** (Line 254) to **Page 15** (Line 555) |
| b. Details of the process measures and outcomes. | **Page 7** (Line 254) to **Page 15** (Line 555) |
| c. Contextual elements that interacted with the intervention(s). | **Page 7** (Line 254) to **Page 19** (Line 555) |
| d. Observed associations between outcomes, interventions and relevant contextual elements. | **Page 7** (Line 254) to **Page 15** (Line 555) |
| e. Unintended consequences such as unexpected benefits, problems, failures or costs associated with the intervention(s). | Not applicable |
| f. Details about missing data. | Not applicable |
|  |  |
| **Discussion: What does it mean?** | **Page 7** (Line 253) to **Page 15** (Line 554) |
| 14. **Summary** | √ |
| a. Key findings, including relevance to the rationale and specific aims. | **Page 3** (Line 100-102) |
| b. Particular strengths of the project. | **Page 16** (Line 572) |
|  |  |
| 15. **Interpretation** | Not applicable |
| a. Nature of the association between the intervention(s) and the outcomes. |  |
| b. Comparison of results with findings from other publications. |  |
| c. Impact of the project on people and systems. |  |
| d. Reasons for any differences between observed and anticipated outcomes, including the influence of context. |  |
| e. Costs and strategic trade-offs, including opportunity costs. |  |
|  |  |
| 16. **Limitations** | √ |
| a. Limits to the generalisability of the work. | **Page 16** (Line 570 to Line 573) |
| b. Factors that might have limited internal validity such as confounding, bias or imprecision in the design, methods, measurement or analysis. | Not applicable |
| c. Efforts made to minimise and adjust for limitations. | Not applicable |
|  |  |
| **Conclusions** | **Page 15** Line 556 |
| a. Usefulness of the work. | Page 15 (Line 557 to Line 558) |
| b. Sustainability. | Page 15 (Line561) to Page 16 (Line 570) |
| c. Potential for spread to other contexts. | Page 16 (Line 573-574) |
| d. Implications for practice and for further study in the field. | Page 16 (Line 573 to Line 574) |
| e. Suggested next steps. | Page 16 (Line 573 to Line 574) |
| **Other information** | √ |
| 18. **Funding** - Sources of funding that supported this work. Role, if any, of the funding organisation in the design, implementation, interpretation and reporting. | Not applicable |
|  |  |
|  |  |
|  |  |
|  |  |
| *Ogrinc G, et al. BMJ Qual Saf 2015;0:1–7. doi:10.1136/bmjqs-2015-004411* |  |
| *Downloaded from http://qualitysafety.bmj.com/ on January 2, 2017* |  |
