# Supplementary material for: Assessment of rational use of antimicrobials: a cross-sectional study among people of Nepal
Source: Ann Med Surg (Lond). 2023 May 24;85(7):3372–80. doi: 10.1097/MS9.0000000000000925 (PMC10328652; doi:10.1097/MS9.0000000000000925)
Supplement: Supplementary file 2 [file ms9-85-3372-s002.docx]

**Supplementary Data File**

**In-silico approach to design effective antiviral drugs against SARS-CoV-2 and SARS-CoV-1 from reported phytochemicals**

***Table S1: Reported 40 Phytochemicals against SARS-CoV-2 and SARS-CoV-1***

| Name | Type | References |
| --- | --- | --- |
| Glabridin | Hydroxyisoflavans | (Islam et al., 2020) |
| 3-O-Feruloylquinic acid | Quinic acid | (Alabboud & Javadmanesh, 2020) |
| (-)-Epicatechin | Catechin | (Pandey & Verma, 2020) |
| (-)-Catechin | (-)-enantiomer of catechin | (Bhushan Mishra et al., 2020) |
| Rhein | Anthracenecarboxylic acids | (Ho et al., 2007) |
| Quercetin | Polyphenolic flavonoid | (Abian et al., 2020) |
| Psoralidin | Phenolic compoundtans | (Mani et al., 2020) |
| Tryptanthrin | Alkaloid | (Narkhede et al., 2020) |
| Lycorine | Indolizidine alkaloid | (Zhang et al., 2020) |
| Scutellarein | flavones | (Sharma & Shanavas, 2020) |
| Ladanein | Flavone | (Lin et al., 2014) |
| Daidzin | Isoflavonoid o-glycosides | (A, 2020) |
| Cryptotanshinone | Diterpenoid | (Boozari & Hosseinzadeh, 2020) |
| Broussochalcone A | Chalcone | (Ghosh et al., 2020) |
| 4'-Hydroxyisolonchocarpin | Carpin | (Park et al., 2017) |
| 3'-(3-methylbut-2-enyl)-3',4',7-trihydroxyflavane | Diphosphate | (Ghosh et al., 2020) |
| Bonducellpin D | Diterpenoid | (Gurung et al., 2020) |
| Bonducellpin C | Diterpenoid | (Gurung et al., 2020) |
| 5-Hydroxy-7,8-dimethoxy (2R)-flavanone-5-o-beta-D-glucopyranoside | homoisoflavonoids | (Brahmachari, 2008) |
| Luteoforol | Pentahydroxyflavan and a leucoanthocyanidin | (Gurung et al., 2020) |
| Hypericin | Anthraquinone | (Islam et al., 2020) |
| Cyanidin 3-O-glucoside | Anthocyanidin-3-o-glycosides | (Kodchakorn et al., 2020) |
| Baicalin | Glycosyloxyflavone | (Jo et al., 2020) |
| (-)-Epigallocatechin gallate | Trihydroxybenzoate | (Maiti & Banerjee, 2020) |
| Silibinin | Flavonolignan | (Kumar et al., 2020) |
| Cassiaoccidentalin A | Flavone | (Ul Qamar et al., 2017) |
| Cassiaoccidentalin B | Flavone | (Ul Qamar et al., 2017) |
| Cassiaoccidentalin C | Flavone | (Ul Qamar et al., 2017) |
| (-)-Gallocatechin gallate | Catechin. | (Takeda et al., 2020) |
| Quercetin 3-glucoside-7-rhamnoside | Glucoside derivative of quercetin | (Russo et al., 2020) |
| Silvestrol | Flavagline | (Boozari & Hosseinzadeh, 2020) |
| Saikosaponin B2 | Plant glycoside | (Mani et al., 2020) |
| Glycyrrhizic acid | Triterpene saponins | (Vardhan & Sahoo, 2020) |
| Hesperidin | Disaccharide derivative | (Bellavite & Donzelli, 2020) |
| Naringin | flavonoid-7-o-glycosides | (Chikhale et al., 2020) |
| Diosmin | Glycosyloxyflavone | (Haggag et al., 2020) |
| Guaijaverin | 3-O-arabinoside | (Azim et al., 2020) |
| Punicalin | Ellagitannin | (Peer-reviewed, 2020) |
| 1,3,4,5-tetra-O-galloylquinic acid | Quinic acid | (Orhan & Senol Deniz, 2020) |
| Beta-sitosterol | Stigmastanes | (Wang et al., 2020) |

***Table S2: Physiochemical parameters of reported phytochemicals***

| Name | MW | Rotatble Bond | HBA | HBD | TPSA | XLOGP3 | ESOL Log S | ESOL Class | Lipinski violations | Bioavailability Score |
| --- | --- | --- | --- | --- | --- | --- | --- | --- | --- | --- |
| Glabridin | 446.36 | 1 | 4 | 2 | 58.92 | 3.89 | -3.41 | Moderately soluble | 0 | 0.55 |
| 3-O-  Feruloylquinicacid- | 482.44 | 6 | 9 | 5 | 153.75 | -0.1 | -4.14 | Very soluble | 0 | 0.11 |
| Epicatechin- | 368.34 | 1 | 6 | 5 | 110.38 | 0.36 | -1.84 | Soluble | 0 | 0.55 |
| Catechin | 290.27 | 1 | 6 | 5 | 110.38 | 0.36 | -2.22 | Soluble | 0 | 0.55 |
| Rhein | 284.22 | 1 | 6 | 3 | 111.9 | 2.23 | -3.36 | Soluble | 0 | 0.56 |
| Quercetin | 302.24 | 1 | 7 | 5 | 131.36 | 1.54 | -3.16 | Soluble | 0 | 0.55 |
| Psoralidin | 336.34 | 2 | 5 | 2 | 83.81 | 4.69 | -5.25 | Moderately soluble | 0 | 0.55 |
| tryptanthrin | 248.24 | 0 | 3 | 0 | 51.96 | 2.05 | -3.29 | Soluble | 0 | 0.55 |
| Lycorine | 287.31 | 0 | 5 | 2 | 62.16 | -0.02 | -1.82 | Very soluble | 0 | 0.55 |
| Scutellarein | 286.24 | 1 | 6 | 4 | 111.13 | 2.66 | -3.79 | Soluble | 0 | 0.55 |
| Griffithsin/**Ladanein** | 314.29 | 3 | 6 | 2 | 89.13 | 3.32 | -4.2 | Moderately soluble | 0 | 0.55 |
| Daidzin | 416.38 | 4 | 9 | 5 | 149.82 | 0.67 | -2.97 | Soluble | 0 | 0.55 |
| Cryptotanshinone | 296.36 | 0 | 3 | 0 | 43.37 | 3.8 | -4.27 | Moderately soluble | 0 | 0.85 |
| BrussochalconeA | 340.37 | 5 | 5 | 4 | 97.99 | 4.75 | -4.97 | Moderately soluble | 0 | 0.55 |
| 4-hydroxyisolonchocarpin | 322.35 | 1 | 4 | 1 | 55.76 | 3.47 | -4.33 | Moderately soluble | 0 | 0.55 |
| 3'-(3-methylbut-2-enyl)-3',4',7-trihydroxyflavane | 328.4 | 3 | 4 | 3 | 69.92 | 3.29 | -3.94 | Soluble | 0 | 0.55 |
| Bonducellpin D | 404.45 | 2 | 7 | 2 | 106.2 | 1.88 | -3.53 | Soluble | 0 | 0.55 |
| Bonducellpin C | 420.5 | 4 | 7 | 2 | 106.2 | 2.39 | -3.81 | Soluble | 0 | 0.55 |
| 5-Hydroxy-7,8-dimethoxy (2R)-flavanone-5-o-beta-D-glucopyranoside | 462.45 | 6 | 10 | 4 | 144.14 | 0.69 | -3.01 | Soluble | 0 | 0.55 |
| Luteoforol | 290.27 | 1 | 6 | 5 | 110.38 | 1.28 | -2.8 | Soluble | 0 | 0.55 |
| Hypericin | 504.44 | 0 | 8 | 6 | 155.52 | 5.71 | -6.99 | Poorly soluble | 2 | 0.17 |
| cyanidin3-glucoside | 504.44 | 4 | 11 | 8 | 193.44 | -1.03 | -6.99 | Soluble | 2 | 0.17 |
| Baicalin | 449.38 | 4 | 11 | 6 | 187.12 | 1.11 | -2.08 | Soluble | 2 | 0.11 |
| (-)-Epigallocatechingallate | 324.37 | 4 | 11 | 8 | 197.37 | 1.17 | -4.61 | Soluble | 2 | 0.17 |
| silibinin | 458.37 | 4 | 10 | 5 | 155.14 | 1.9 | -3.56 | Moderately soluble | 0 | 0.55 |
| CassiaoccidentalinA- | 270.24 | 4 | 13 | 7 | 216.58 | 0.04 | -3.04 | Soluble | 3 | 0.17 |
| CassiaoccidentalinB- | 560.5 | 4 | 14 | 8 | 236.81 | -0.32 | -3.37 | Soluble | 3 | 0.17 |
| CassiaoccidentalinC- | 576.5 | 5 | 14 | 7 | 225.81 | 0.01 | -3.24 | Soluble | 3 | 0.17 |
| (-)Gallocatechingallate- | 458.37 | 4 | 11 | 8 | 197.37 | 1.17 | -3.56 | Soluble | 2 | 0.17 |
| Quercetin-3-glucoside-7-rhamnoside | 610.52 | 6 | 16 | 10 | 269.43 | 0.01 | -3.51 | Soluble | 3 | 0.17 |
| Silvestrol | 654.66 | 11 | 13 | 4 | 171.83 | 1.64 | -4.49 | Moderately soluble | 2 | 0.17 |
| SaikosaponinB2 | 780.98 | 7 | 13 | 9 | 218.99 | 2.53 | -5.81 | Moderately soluble | 3 | 0.17 |
| Liquorice | 822.93 | 7 | 16 | 8 | 267.04 | 2.8 | -6.24 | Poorly soluble | 3 | 0.11 |
| Hesperidin | 610.56 | 7 | 15 | 8 | 234.29 | -0.14 | -3.28 | Soluble | 3 | 0.17 |
| Narigin | 580.53 | 6 | 14 | 8 | 225.06 | -0.44 | -2.98 | Soluble | 3 | 0.17 |
| Diosmin | 608.54 | 7 | 15 | 8 | 238.2 | 0.14 | -3.51 | Soluble | 3 | 0.17 |
| Guaijaverin | 434.35 | 3 | 11 | 7 | 190.28 | 0.43 | -2.99 | Soluble | 2 | 0.17 |
| Punicalin | 782.53 | 0 | 22 | 13 | 385.24 | -0.29 | -4.88 | Moderately soluble | 3 | 0.17 |
| 1,3,4,5-Tetra-O-galloylquinic acid | 800.58 | 13 | 22 | 13 | 385.26 | 2.3 | -5.71 | Moderately soluble | 3 | 0.11 |
| Beta-Sitosterol | 414.71 | 6 | 1 | 1 | 20.23 | 9.34 | -7.9 | Poorly soluble | 1 | 0.55 |

***Table S3: Docking results of set-B phytochemicals with the main protease of SARS-CoV-2 and SARS-CoV-1***

| SARS-CoV-2 | | SARS-CoV-1 | |
| --- | --- | --- | --- |
| Ligand | Binding Affinity | Ligand | Binding Affinity |
| Hypericin | -10.6 | Hypericin | -9.7 |
| Diosmin | -8.9 | Hesperidin | -9 |
| (-)-Epigallocatechingallate | -8.9 | (-)-Epigallocatechingallate | -8.8 |
| (-)-Gallocatechingallate- | -8.8 | (-)-Gallocatechingallate- | -8.7 |
| Guaijaverin | -8.7 | Diosmin | -8.4 |
| Narigin | -8.5 | Silibinin | -8.4 |
| Hesperidin | -8.4 | 1,3,4,5-Tetra-O-galloylquinic acid | -8.4 |
| CassiaoccidentalinA- | -8.4 | CassiaoccidentalinA- | -8.3 |
| CassiaoccidentalinB- | -8.2 | Narigin | -8.3 |
| Punicalin | -8.2 | CassiaoccidentalinC- | -8.2 |
| 1,3,4,5-Tetra-O-galloylquinic acid | -8.2 | SaikosaponinB2 | -8.1 |
| Silibinin | -8 | Quercetin-3-glucoside-7-rhamnoside | -8 |
| Baicalin | -8 | CassiaoccidentalinB- | -8 |
| Liquorice | -7.9 | Liquorice | -8 |
| CassiaoccidentalinC- | -7.8 | Baicalin | -7.9 |
| Quercetin-3-glucoside-7-rhamnoside | -7.5 | Guaijaverin | -7.6 |
| SaikosaponinB2 | -7.5 | Silvestrol | -7.3 |
| Silvestrol | -7.1 | Punicalin | -7.2 |
| Cyanidin3-glucoside | -7 | Cyanidin3-glucoside | -6.5 |
| beta-Sitosterol | -6.4 | beta-Sitosterol | -6 |

***Table S4: Noncovalent interactions of selected five phytochemicals (set-B) with the main protease of SARS-CoV-2 and SARS-CoV-1 (pose predicted by AutoDock Vina)***

| SARS-CoV-2 | | | | SARS-CoV-1 | | | |
| --- | --- | --- | --- | --- | --- | --- | --- |
| **Hypericin** | | | | **Hypericin** | | | |
| Residues | Distance  (Å) | Bond  Category | Bond  Types | Residues | Distance  (Å) | Bond  Category | Bond  Types |
| GLU166 | 2.8852 | Hydrogen Bond | Conventional  Hydrogen  Bond | ARG188 | 2.84091 | Hydrogen Bond | Conventional Hydrogen Bond |
| LEU141 | 2.50151 | Hydrogen Bond | Conventional  Hydrogen  Bond | GLN189 | 2.75289 | Hydrogen Bond | Conventional Hydrogen Bond |
| CYS145 | 2.52639 | Hydrogen Bond | Conventional  Hydrogen  Bond | ARG188 | 2.81295 | Hydrogen Bond | Conventional Hydrogen Bond |
| GLU166 | 3.0828 | Hydrogen Bond | Pi-Donor Hydrogen Bond | ASN142 | 2.84484 | Hydrogen Bond | Conventional Hydrogen Bond |
| GLU166 | 2.71383 | Hydrophobic | Pi-Sigma | LEU141 | 3.02502 | Hydrogen Bond | Carbon Hydrogen Bond |
| GLN189 | 2.49583 | Hydrophobic | Pi-Sigma | GLN189 | 2.48215 | Hydrogen Bond | Carbon Hydrogen Bond |
| MET165 | 4.24459 | Hydrophobic | Alkyl | GLU166 | 3.87963 | Electrostatic | Pi-Anion |
| MET165 | 4.36954 | Hydrophobic | Pi-Alkyl | GLU166 | 4.69397 | Electrostatic | Pi-Anion |
| CYS145 | 4.99003 | Hydrophobic | Pi-Alkyl | GLU166 | 2.94939 | Hydrogen Bond | Pi-Donor Hydrogen Bond |
| Diosmin | | | | CYS145 | 4.33958 | Hydrophobic | Alkyl |
| GLU166 | 2.73174 | Hydrogen Bond | Conventional Hydrogen Bond | CYS145 | 4.15462 | Hydrophobic | Alkyl |
| ARG188 | 2.92372 | Hydrogen Bond | Conventional Hydrogen Bond | MET165 | 4.868 | Hydrophobic | Pi-Alkyl |
| UNK1 | 2.66364 | Hydrogen Bond | Conventional Hydrogen Bond | MET165 | 5.18466 | Hydrophobic | Pi-Alkyl |
| SER144 | 2.6639 | Hydrogen Bond | Conventional Hydrogen Bond | HIS163 | 4.56871 | Hydrophobic | Pi-Alkyl |
| GLU166 | 2.44894 | Hydrogen Bond | Carbon Hydrogen Bond | **Hesperidin** | | | |
| THR24 | 2.72996 | Hydrogen Bond | Carbon Hydrogen Bond | THR190 | 1.97432 | Hydrogen Bond | Conventional Hydrogen Bond |
| HIS164 | 3.0218 | Hydrogen Bond | Carbon Hydrogen Bond | ASN142 | 2.15567 | Hydrogen Bond | Conventional Hydrogen Bond |
| HIS41 | 2.41699 | Hydrophobic | Pi-Sigma | THR45 | 2.8305 | Hydrogen Bond | Carbon Hydrogen Bond |
| CYS145 | 5.805 | Other | Pi-Sulfur | GLU166 | 2.80367 | Hydrogen Bond | Carbon Hydrogen Bond |
| MET49 | 4.35187 | Hydrophobic | Alkyl | THR45 | 2.74187 | Hydrogen Bond | Carbon Hydrogen Bond |
| CYS145 | 4.68534 | Hydrophobic | Pi-Alkyl | GLY143 | 2.83378 | Hydrogen Bond | Carbon Hydrogen Bond |
| HIS41 | 4.22056 | Hydrophobic | Pi-Alkyl | GLN189 | 2.71776 | Hydrogen Bond | Carbon Hydrogen Bond |
| **(-)-Epigallocatechingallate** | | | | CYS145 | 4.71804 | Hydrophobic | Pi-Alkyl |
| CYS145 | 2.89391 | Hydrogen Bond | Conventional Hydrogen Bond | **(-)-Epigallocatechingallate** | | | |
| TYR54 | 2.57093 | Hydrogen Bond | Conventional Hydrogen Bond | CYS145 | 2.90815 | Hydrogen Bond | Conventional Hydrogen Bond |
| GLU166 | 2.42829 | Hydrogen Bond | Conventional Hydrogen Bond | LEU141 | 2.4024 | Hydrogen Bond | Conventional Hydrogen Bond |
| GLN192 | 2.98647 | Hydrogen Bond | Conventional Hydrogen Bond | GLN192 | 2.99882 | Hydrogen Bond | Conventional Hydrogen Bond |
| GLN189 | 2.45666 | Hydrogen Bond | Carbon Hydrogen Bond | GLU166 | 2.71591 | Hydrogen Bond | Conventional Hydrogen Bond |
| GLN189 | 3.04866 | Hydrogen Bond | Carbon Hydrogen Bond | GLN189 | 2.8296 | Hydrogen Bond | Carbon Hydrogen Bond |
| MET165 | 4.91039 | Other | Pi-Sulfur | GLU166 | 2.97871 | Hydrogen Bond | Carbon Hydrogen Bond |
| HIS41 | 4.5359 | Hydrophobic | Pi-Pi T-shaped | CYS145 | 5.97437 | Other | Pi-Sulfur |
| MET165 | 4.67794 | Hydrophobic | Alkyl | HIS41 | 4.72068 | Hydrophobic | Pi-Pi T-shaped |
| MET49 | 4.65718 | Hydrophobic | Pi-Alkyl | MET165 | 4.45619 | Hydrophobic | Alkyl |
| **(-)-Gallocatechingallate-** | | | | MET49 | 5.28667 | Hydrophobic | Pi-Alkyl |
| PHE140 | 1.85369 | Hydrogen Bond | Conventional Hydrogen Bond | MET165 | 4.77398 | Hydrophobic | Pi-Alkyl |
| HIS163 | 2.7133 | Hydrogen Bond | Conventional Hydrogen Bond | **(-)-Gallocatechingallate** | | | |
| GLN189 | 3.06625 | Hydrogen Bond | Conventional Hydrogen Bond | PHE140 | 1.85369 | Hydrogen Bond | Conventional Hydrogen Bond |
| CYS145 | 5.67136 | Other | Pi-Sulfur | HIS163 | 2.7133 | Hydrogen Bond | Conventional Hydrogen Bond |
| MET49 | 5.03445 | Hydrophobic | Alkyl | GLN189 | 3.06625 | Hydrogen Bond | Conventional Hydrogen Bond |
| MET49 | 4.57308 | Hydrophobic | Pi-Alkyl | CYS145 | 5.67136 | Other | Pi-Sulfur |
| HIS41 | 4.74754 | Hydrophobic | Pi-Alkyl | MET49 | 5.03445 | Hydrophobic | Alkyl |
| Guaijaverin | | | | MET49 | 4.57308 | Hydrophobic | Pi-Alkyl |
| ASN142 | 1.82963 | Hydrogen Bond | Conventional Hydrogen Bond | HIS41 | 4.74754 | Hydrophobic | Pi-Alkyl |
| THR26 | 2.52158 | Hydrogen Bond | Conventional Hydrogen Bond | Diosmin | | | |
| ASP187 | 2.39999 | Hydrogen Bond | Conventional Hydrogen Bond | GLN189 | 2.88017 | Hydrogen Bond | Conventional Hydrogen Bond |
| GLY143 | 2.81869 | Hydrogen Bond | Conventional Hydrogen Bond | GLN189 | 2.63277 | Hydrogen Bond | Conventional Hydrogen Bond |
| SER144 | 2.81246 | Hydrogen Bond | Conventional Hydrogen Bond | THR24 | 2.67173 | Hydrogen Bond | Conventional Hydrogen Bond |
| HIS163 | 2.53889 | Hydrogen Bond | Carbon Hydrogen Bond | THR26 | 2.17787 | Hydrogen Bond | Conventional Hydrogen Bond |
| ASN142 | 2.58065 | Hydrogen Bond | Carbon Hydrogen Bond | GLN189 | 2.57784 | Hydrogen Bond | Conventional Hydrogen Bond |
| MET165 | 3.04808 | Hydrogen Bond | Carbon Hydrogen Bond | THR26 | 2.80698 | Hydrogen Bond | Carbon Hydrogen Bond |
| MET165 | 2.33941 | Hydrogen Bond | Carbon Hydrogen Bond | THR190 | 2.70803 | Hydrogen Bond | Carbon Hydrogen Bond |
| ARG188 | 2.74884 | Hydrogen Bond | Carbon Hydrogen Bond | CYS145 | 3.4987 | Other | Pi-Sulfur |
| CYS145 | 4.97218 | Other | Pi-Sulfur | CYS145 | 5.00365 | Hydrophobic | Pi-Alkyl |
| MET49 | 4.88415 | Hydrophobic | Pi-Alkyl | MET165 | 5.41085 | Hydrophobic | Pi-Alkyl |
| MET49 | 4.39099 | Hydrophobic | Pi-Alkyl |  |  |  |  |

***Table S5. Multiple Linear Regression Model Training set and Test set Data***

| Training set (25) | | | | | Test set (15) | | |
| --- | --- | --- | --- | --- | --- | --- | --- |
| Name | Y(Obs) | Y(Cal) | Y(res) | St. Dev Res | Compound | B. A | Predicted |
| CassiaoccidentalinA | -8.4 | -8.188 | -0.212 | -0.671 | Quercetin-3-glucoside-7-rhamnoside | -7.5 | -8.66 |
| Cassiaoccidentalin | -8.2 | -8.319 | 0.119 | 0.377 | Quercetin | -7.5 | -8.21 |
| Cassiaoccidentalin | -7.8 | -8.12 | 0.32 | 1.015 | Psoralidin | -7.5 | -8.27 |
| (-)-Gallocatechingallate | -8.8 | -8.375 | -0.425 | -1.346 | tryptanthrin | -7.2 | -6.85 |
| Lycorine | -7.3 | -7.132 | -0.168 | -0.531 | SaikosaponinB2 | -7.5 | -8.4 |
| Scutellarein | -8 | -8.167 | 0.167 | 0.529 | Hesperidin | -8.4 | -7.98 |
| Silvestrol | -7.1 | -7.173 | 0.073 | 0.231 | Narigin | -8.5 | -7.99 |
| Liquorice | -7.9 | -7.945 | 0.045 | 0.144 | Diosmin | -8.9 | -8.15 |
| Guaijaverin | -8.7 | -8.247 | -0.453 | -1.436 | beta-Sitosterol | -6.4 | -7.47 |
| Punicalin | -8.2 | -8.2 | 0 | 0 | Griffithsin/ladanein | -7.3 | -7.73 |
| 1,3,4,5-Tetra-O-galloylquinic acid | -8.2 | -8.378 | 0.178 | 0.565 | BrussochalconeA | -8 | -7.62 |
| Daidzin | -7.6 | -7.691 | 0.091 | 0.288 | 4-hydroxyisolonchocarpin | -8.1 | -7.77 |
| Cryptotanshinone | -7.4 | -7.447 | 0.047 | 0.149 | Bonducellpin D | -6.7 | -7.23 |
| 3'-(3-methylbut-2-enyl)-3',4',7-trihydroxyflavane | -7.4 | -7.39 | -0.01 | -0.033 | Luteoforol | -7.6 | -8.12 |
| Bonducellpin C | -7.3 | -6.983 | -0.317 | -1.006 | (-)-Epigallocatechingallate | -8.9 | -8.51 |
| 5-Hydroxy-7,8-dimethoxy (2R)-flavanone-5-o-beta-D-glucopyranoside | -7.3 | -7.206 | -0.094 | -0.299 |  |  |  |
| Hypericin | -10.6 | -10.324 | -0.276 | -0.874 |  |  |  |
| cyanidin3-glucoside | -7 | -7.103 | 0.103 | 0.328 |  |  |  |
| Baicalin | -8 | -7.966 | -0.034 | -0.107 |  |  |  |
| silibinin | -8 | -8.225 | 0.225 | 0.714 |  |  |  |
| Glabridin | -7.9 | -8.098 | 0.198 | 0.627 |  |  |  |
| 3-O-Feruloylquinicacid | -6.9 | -6.486 | -0.414 | -1.311 |  |  |  |
| Catechin | -7.8 | -7.753 | -0.047 | -0.148 |  |  |  |
| Rhein | -7.3 | -7.529 | 0.229 | 0.727 |  |  |  |
| Epicatechin | -7.1 | -7.753 | 0.653 | 2.07 |  |  |  |


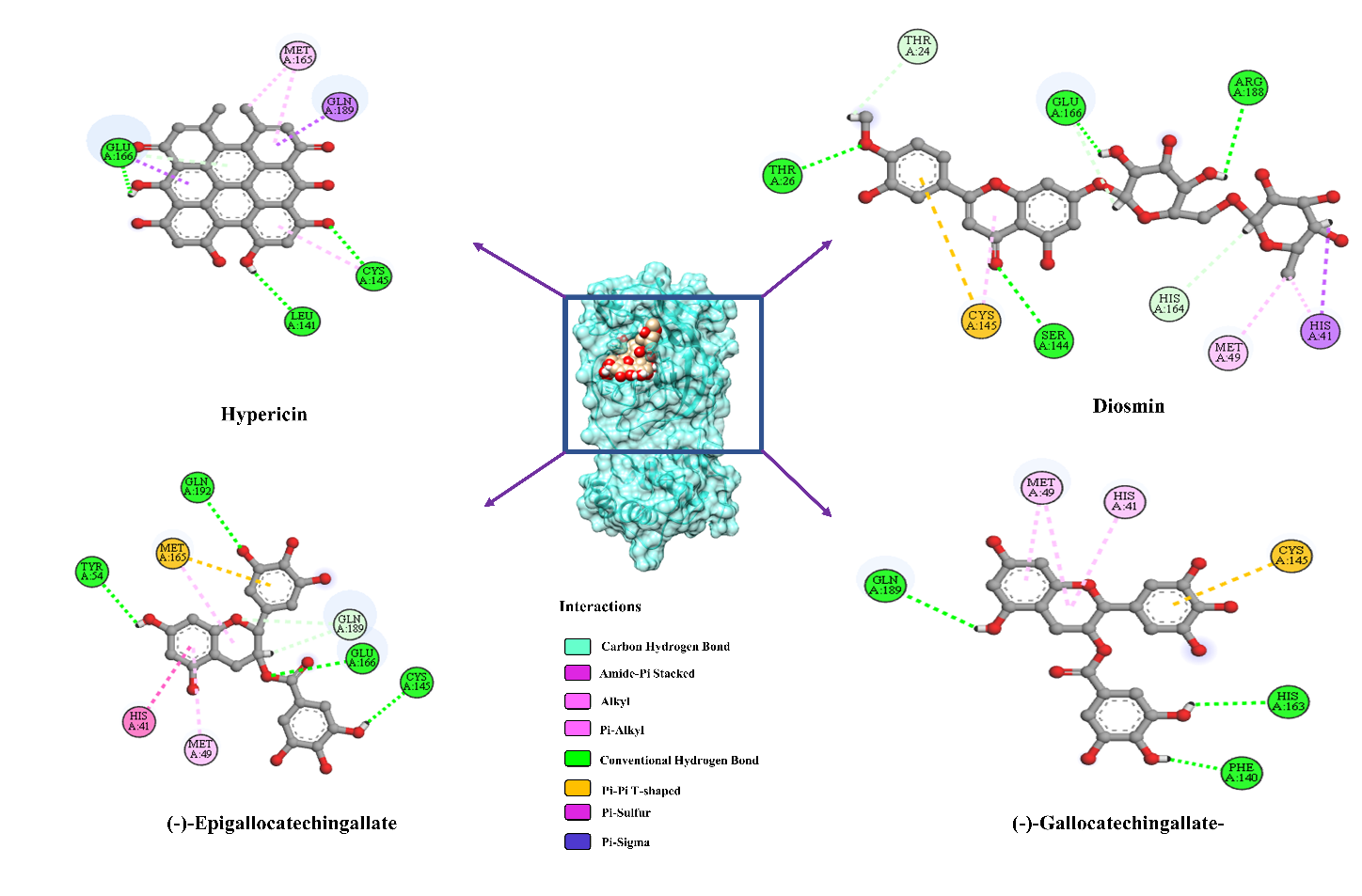


***Figure S1: Interaction residues of the top four phytochemicals (set-B) with SARS-CoV-2 main protease.***


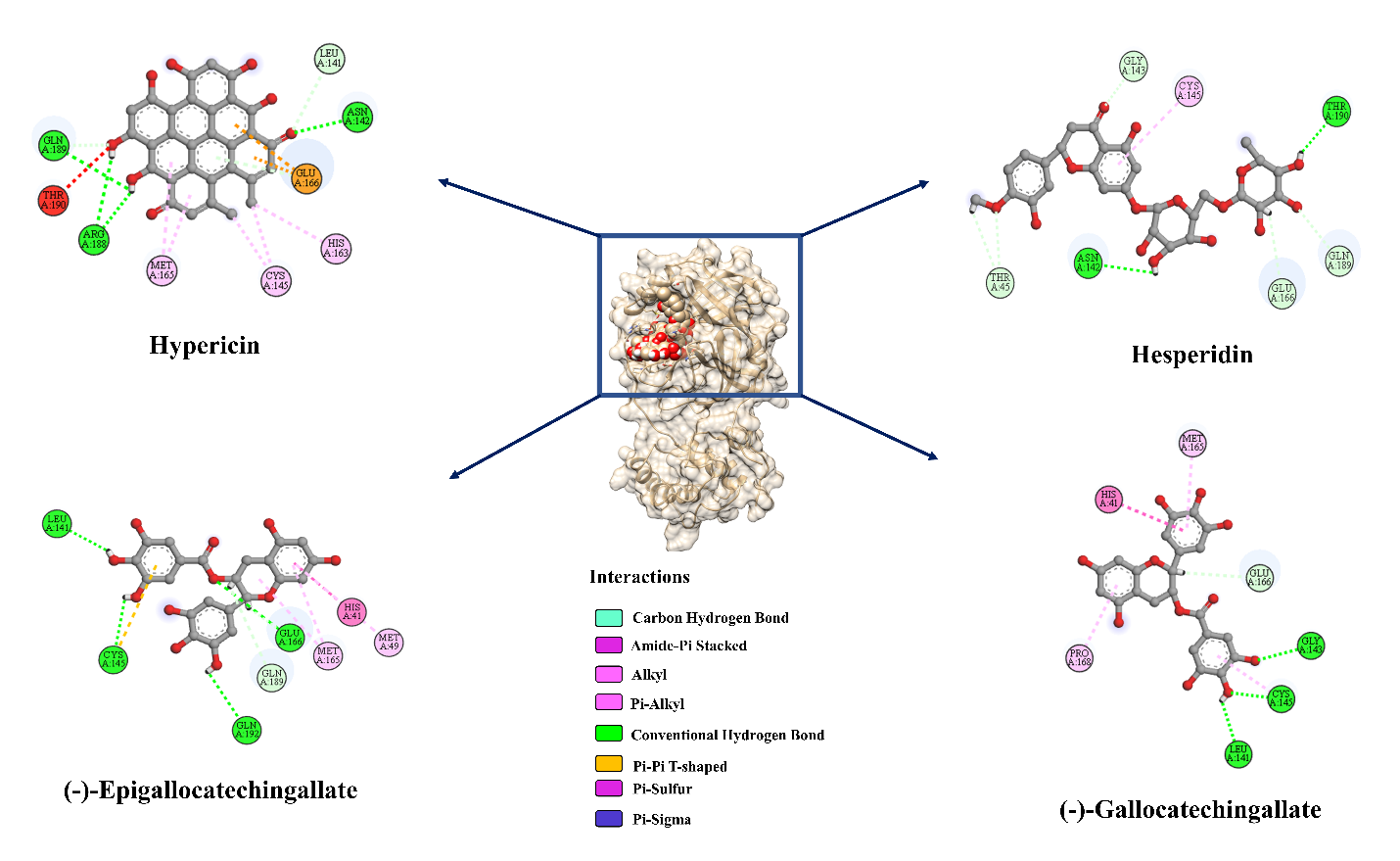


***Figure S2: Interaction residues of the top four phytochemicals (set-B) with SARS-CoV-1 main protease.***


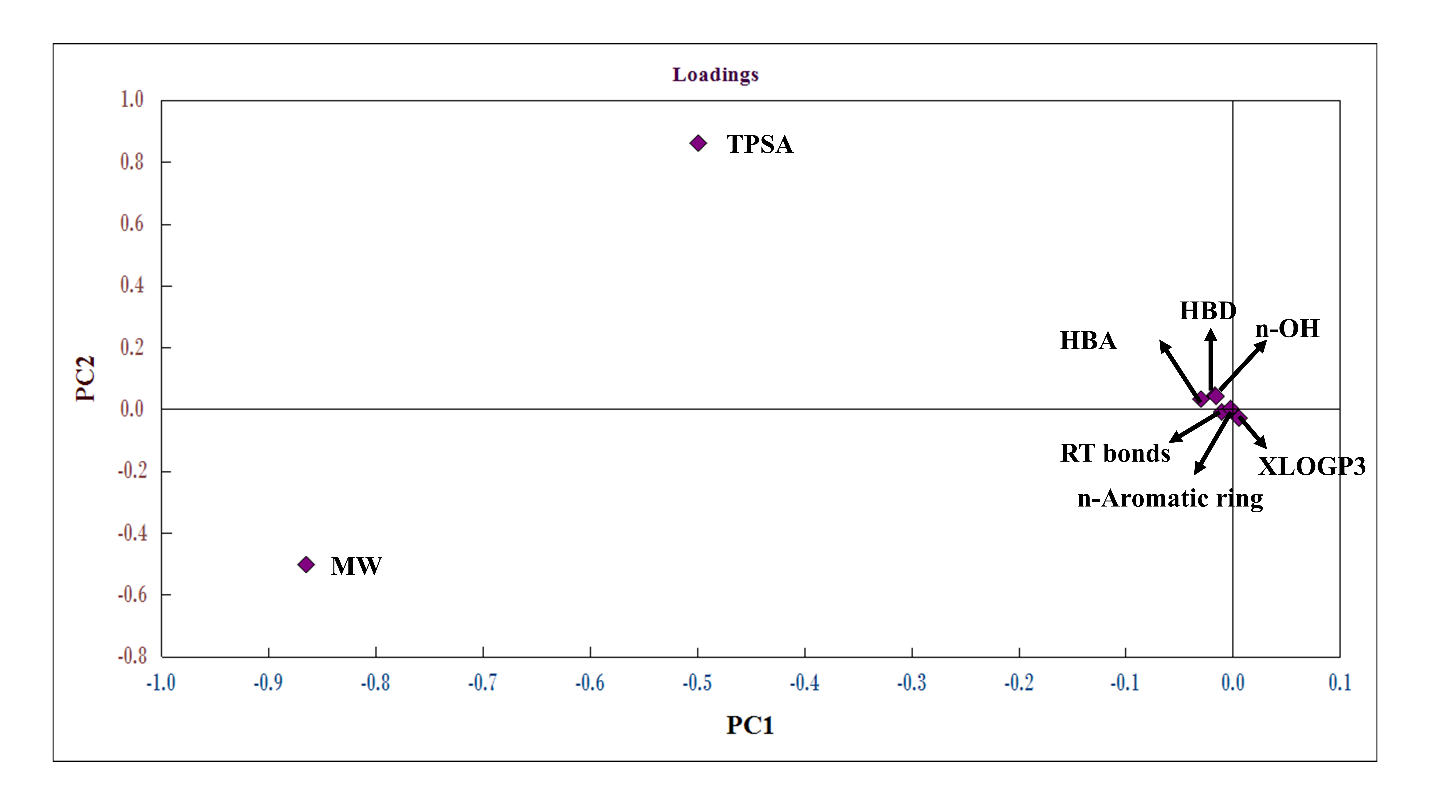


***Figure S3: Loading plot of PCA analysis of 25 phytochemicals***

Protein-Drug dynamic movement video:

1. 4-hydroxyisolonchocarpin against SARS-CoV-1
2. 4-hydroxyisolonchocarpin against SARS-COV-2
3. Brussochalcone A against SARS-CoV-1
4. Brussochalcone A against SARS-CoV-2

References:

A, E. A. (2020). pt e cr. *Journal of Biomolecular Structure and Dynamics*, *0*(0), 000. https://doi.org/10.1080/07391102.2020.1761881

Abian, O., Ortega-Alarcon, D., Jimenez-Alesanco, A., Ceballos-Laita, L., Vega, S., Reyburn, H. T., Rizzuti, B., & Velazquez-Campoy, A. (2020). Structural stability of SARS-CoV-2 3CLpro and identification of quercetin as an inhibitor by experimental screening. *International Journal of Biological Macromolecules*, *164*, 1693–1703. https://doi.org/10.1016/j.ijbiomac.2020.07.235

Alabboud, M., & Javadmanesh, A. (2020). *In silico study of various antiviral drugs , vitamins , and natural substances as potential binding compounds with SARS-CoV-2 main protease*. *April*. https://doi.org/10.30493/DLS.2020.225404

Azim, K. F., Ahmed, S. R., Banik, A., Khan, M. M. R., Deb, A., & Somana, S. R. (2020). Screening and druggability analysis of some plant metabolites against SARS-CoV-2: An integrative computational approach. *Informatics in Medicine Unlocked*, *20*, 100367. https://doi.org/10.1016/j.imu.2020.100367

Bellavite, P., & Donzelli, A. (2020). Hesperidin and SARS-CoV-2: New light on the healthy function of citrus fruits. *Antioxidants*, *9*(8), 1–18. https://doi.org/10.3390/antiox9080742

Bhushan Mishra, C., Pandey, P., Datta Sharma, R., Kumar Mongre, R., Lynn, A. M., Prasad, R., Jeon, R., & Prakash, A. (2020). *Discovery of Natural Phenol Catechin as a Multitargeted Agent Against SARS-CoV-2 For the Plausible Therapy of COVID-19*. 1–33. https://doi.org/10.26434/chemrxiv.12752402.v1

Boozari, M., & Hosseinzadeh, H. (2020). Natural products for COVID-19 prevention and treatment regarding to previous coronavirus infections and novel studies. *Phytotherapy Research*, *August*, 1–13. https://doi.org/10.1002/ptr.6873

Brahmachari, G. (2008). Naturally occurring flavanones: An overview. *Natural Product Communications*, *3*(8), 1337–1354. https://doi.org/10.1177/1934578x0800300820

Chikhale, R. V., Gupta, V. K., Eldesoky, G. E., Wabaidur, S. M., Patil, S. A., & Islam, M. A. (2020). Identification of potential anti-TMPRSS2 natural products through homology modelling, virtual screening and molecular dynamics simulation studies. *Journal of Biomolecular Structure and Dynamics*, *0*(0), 1–16. https://doi.org/10.1080/07391102.2020.1798813

Ghosh, R., Chakraborty, A., Biswas, A., & Chowdhuri, S. (2020). Identification of polyphenols from Broussonetia papyrifera as SARS CoV-2 main protease inhibitors using in silico docking and molecular dynamics simulation approaches. *Journal of Biomolecular Structure and Dynamics*, *0*(0), 1–14. https://doi.org/10.1080/07391102.2020.1802347

Gurung, A. B., Ali, M. A., Lee, J., Farah, M. A., & Al-Anazi, K. M. (2020). Unravelling lead antiviral phytochemicals for the inhibition of SARS-CoV-2 Mpro enzyme through in silico approach. *Life Sciences*, *255*, 117831. https://doi.org/10.1016/j.lfs.2020.117831

Haggag, Y. A., El-Ashmawy, N. E., & Okasha, K. M. (2020). Is hesperidin essential for prophylaxis and treatment of COVID-19 Infection? *Medical Hypotheses*, *144*, 109957. https://doi.org/10.1016/j.mehy.2020.109957

Ho, T. Y., Wu, S. L., Chen, J. C., Li, C. C., & Hsiang, C. Y. (2007). Emodin blocks the SARS coronavirus spike protein and angiotensin-converting enzyme 2 interaction. *Antiviral Research*, *74*(2), 92–101. https://doi.org/10.1016/j.antiviral.2006.04.014

Islam, R., Parves, M. R., Paul, A. S., Uddin, N., Rahman, M. S., Mamun, A. Al, Hossain, M. N., Ali, M. A., & Halim, M. A. (2020). A molecular modeling approach to identify effective antiviral phytochemicals against the main protease of SARS-CoV-2. *Journal of Biomolecular Structure and Dynamics*, *0*(0), 000. https://doi.org/10.1080/07391102.2020.1761883

Jo, S., Kim, S., Kim, D. Y., Kim, M. S., & Shin, D. H. (2020). Flavonoids with inhibitory activity against SARS-CoV-2 3CLpro. *Journal of Enzyme Inhibition and Medicinal Chemistry*, *35*(1), 1539–1544. https://doi.org/10.1080/14756366.2020.1801672

Kodchakorn, K., Poovorawan, Y., Suwannakarn, K., & Kongtawelert, P. (2020). Molecular modelling investigation for drugs and nutraceuticals against protease of SARS-CoV-2. *Journal of Molecular Graphics and Modelling*, *101*, 107717. https://doi.org/10.1016/j.jmgm.2020.107717

Kumar, S., Kashyap, P., Chowdhury, S., Kumar, S., Panwar, A., & Kumar, A. (2020). Identification of phytochemicals as potential therapeutic agents that binds to Nsp15 protein target of coronavirus (SARS-CoV-2) that are capable of inhibiting virus replication. *Phytomedicine*, *August*, 153317. https://doi.org/10.1016/j.phymed.2020.153317

Lin, L. T., Hsu, W. C., & Lin, C. C. (2014). Antiviral natural products and herbal medicines. *Journal of Traditional and Complementary Medicine*, *4*(1), 24–35. https://doi.org/10.4103/2225-4110.124335

Maiti, S., & Banerjee, A. (2020). Epigallocatechin gallate and theaflavin gallate interaction in SARS-CoV-2 spike-protein central channel with reference to the hydroxychloroquine interaction: Bioinformatics and molecular docking study. *Drug Development Research*, *April*, 1–11. https://doi.org/10.1002/ddr.21730

Mani, J. S., Johnson, J. B., Steel, J. C., Broszczak, D. A., Neilsen, P. M., Walsh, K. B., & Naiker, M. (2020). Natural product-derived phytochemicals as potential agents against coronaviruses : A review. *Virus Research*, *284*(April), 197989. https://doi.org/10.1016/j.virusres.2020.197989

Narkhede, R. R., Pise, A. V, Cheke, R. S., & Shinde, S. D. (2020). Recognition of Natural Products as Potential Inhibitors of COVID ‑ 19 Main Protease ( Mpro ): In ‑ Silico Evidences. *Natural Products and Bioprospecting*, *0123456789*. https://doi.org/10.1007/s13659-020-00253-1

Orhan, I. E., & Senol Deniz, F. S. (2020). Natural Products as Potential Leads Against Coronaviruses: Could They be Encouraging Structural Models Against SARS-CoV-2? *Natural Products and Bioprospecting*, *10*(4), 171–186. https://doi.org/10.1007/s13659-020-00250-4

Pandey, A. K., & Verma, S. (2020). An in-silico evaluation of dietary components for structural inhibition of SARS-Cov-2 main protease. *Journal of Biomolecular Structure and Dynamics*, *0*(0), 1–7. https://doi.org/10.1080/07391102.2020.1809522

Park, J. Y., Yuk, H. J., Ryu, H. W., Lim, S. H., Kim, K. S., Park, K. H., Ryu, Y. B., & Lee, W. S. (2017). Evaluation of polyphenols from Broussonetia papyrifera as coronavirus protease inhibitors. *Journal of Enzyme Inhibition and Medicinal Chemistry*, *32*(1), 504–512. https://doi.org/10.1080/14756366.2016.1265519

Peer-reviewed, N. O. T. (2020). *© 2020 by the author(s). Distributed under a Creative Commons CC BY license.* *19*(April), 1–37. https://doi.org/10.20944/preprints202003.0277.v1

Russo, M., Moccia, S., Spagnuolo, C., Tedesco, I., & Russo, G. L. (2020). Roles of flavonoids against coronavirus infection. *Chemico-Biological Interactions*, *328*(June), 109211. https://doi.org/10.1016/j.cbi.2020.109211

Sharma, P., & Shanavas, A. (2020). Natural derivatives with dual binding potential against SARS-CoV-2 main protease and human ACE2 possess low oral bioavailability: a brief computational analysis. *Journal of Biomolecular Structure and Dynamics*, *0*(0), 1–12. https://doi.org/10.1080/07391102.2020.1794970

Takeda, Y., Murata, T., Jamsransuren, D., Suganuma, K., Kazami, Y., Batkhuu, J., Badral, D., & Ogawa, H. (2020). Saxifraga spinulosa-derived components rapidly inactivate multiple viruses including SARS-CoV-2. *Viruses*, *12*(7). https://doi.org/10.3390/v12070699

Ul Qamar, M. T., Ashfaq, U. A., Tusleem, K., Mumtaz, A., Tariq, Q., Goheer, A., & Ahmed, B. (2017). In-silico identification and evaluation of plant flavonoids as dengue NS2B/NS3 protease inhibitors using molecular docking and simulation approach. *Pakistan Journal of Pharmaceutical Sciences*, *30*(6), 2119–2137.

Vardhan, S., & Sahoo, S. K. (2020). In silico ADMET and molecular docking study on searching potential inhibitors from limonoids and triterpenoids for COVID-19. *Computers in Biology and Medicine*, *124*(July), 103936. https://doi.org/10.1016/j.compbiomed.2020.103936

Wang, J., Zhang, X., Omarini, A. B., & Li, B. (2020). Virtual screening for functional foods against the main protease of SARS-CoV-2. *Journal of Food Biochemistry*, *August*, 1–11. https://doi.org/10.1111/jfbc.13481

Zhang, Y. N., Zhang, Q. Y., Li, X. D., Xiong, J., Xiao, S. Q., Wang, Z., Zhang, Z. R., Deng, C. L., Yang, X. Lou, Wei, H. P., Yuan, Z. M., Ye, H. Q., & Zhang, B. (2020). Gemcitabine, lycorine and oxysophoridine inhibit novel coronavirus (SARS-CoV-2) in cell culture. *Emerging Microbes and Infections*, *9*(1), 1170–1173. https://doi.org/10.1080/22221751.2020.1772676
